# Reliability of Low-Cost, Sensor-Based Fine Dust Measurement Devices for Monitoring Atmospheric Particulate Matter Concentrations

**DOI:** 10.3390/ijerph16081430

**Published:** 2019-04-22

**Authors:** Eun-Min Cho, Hyung Jin Jeon, Dan Ki Yoon, Si Hyun Park, Hyung Jin Hong, Kil Yong Choi, Heun Woo Cho, Hyo Chang Cheon, Cheol Min Lee

**Affiliations:** 1 Department of Applied Chemistry, College of Applied Science, Kyung Hee University, Yongin 17104, Korea; choeunmin@khu.ac.kr; 2 Korea Environmental Information Center, Korea Environment Institute, Bldg B, 370 Sicheong-daero, Sejong-si 30147, Korea; hjjeon@kei.re.kr; 3 Institute of Risk Assessment, Department of Chemical & Biological Engineering, Seokyeong University, 124 Seogyeong-ro, Seongbuk-gu, Seoul 02173, Korea; ydk0207@skuniv.ac.kr (D.K.Y.); yokkkk@naver.com (S.H.P.); hongdonn01@skuniv.ac.kr (H.J.H.); bestchoi94@naver.com (K.Y.C.); 4 E-Three. Co., Ltd, B-309, Woolim Blue 9 Business Center, Yangcheon-ro 583, Gangseo-gu, Seoul 07547, Korea; jungkil01@hanmail.net (H.W.C.); hccheon@ethree.co.kr (H.C.C.)

**Keywords:** low-cost sensors, sensor evaluation, fine dust, particle pollutants, statistical analysis

## Abstract

Currently, low-cost, sensor-based fine dust measurement devices are commercially available in South Korea. This study evaluated the reliability of three such devices—Yi Shan A4, Plantower PMS7003, and Plantower PMS7003—in comparison to long-term consecutive monitoring systems for discharge and prevention facilities regarding fine dust control. The performance of these devices for concentration intervals over time was examined through real-time comparison using a GRIMM (Model: 11-A, dust spectrometer from Grimm Technologies) as a reference; this included a correction factor (C-Factor), calculated by a gravimetric method and an equivalence test. For comparison, the reference and target devices were installed in a chamber with fine dust concentrations of 2 µg/m^3^, with temperature and humidity maintained at 20 °C and 40%, respectively. The fine particulate matter (PM)_2.5_ concentrations were classified into five intervals: ≤40 µg/m^3^, 40–80 µg/m^3^, 80–120 µg/m^3^, 120–160 µg/m^3^, and 200–230 µg/m^3^. Statistical analysis was performed using data obtained from national stations for monitoring and controlling fine dust released from facilities under high fine dust loading conditions. The results showed that the measurements of all target devices, which were corrected according to the reference device, provided accurate values at PM_2.5_ concentrations of ≥40 µg/m^3^. The statistical analysis results suggest that the evaluated devices are more reliable than the conventional numerical-analysis-based monitoring system

## 1. Introduction

Both human life and the environment are significantly affected by air quality. Numerous studies have attempted to reveal the relationship between air pollution and the quality of life and health. Air pollution deteriorates pulmonary functions, aggravates respiratory and cardiovascular problems, and increases asthma rates [1]. In addition to health problems, air pollution is also detrimental to vegetation [2], visibility, the global climate, and many other factors that impact human life. 

Since the enactment of the Clean Air Conservation Act in South Korea in 1990, the Ministry of Environment has revised the act multiple times to ensure appropriate and sustainable conservation of the atmosphere and prevent detrimental impacts of air pollution on human health and the environment, and to maintain a healthy and clean environment. In compliance with Article 3, specifying regular measurements, monitoring networks have been installed since 1973 to measure air quality in accordance with the Enforcement Rule of the Ministry. Currently, 11 types of air pollution monitoring networks (urban area, roadside, acid deposition, national background, suburb, airborne heavy metals, air pollutants, photochemical air pollutants, earth, PM_2.5_, and intensive monitoring) are operated by the Ministry of Environment and local governments. In total, 510 stations have been installed in 95 cities and counties of the country [3]. However, air pollutants show strong spatial variability; their concentrations vary according to region, and the variability of each pollutant is also very high because of diverse factors, such as pollutant type and atmospheric conditions [4,5,6,7]. For this reason, the reliability of data obtained from the current air pollution networks for air quality monitoring in local areas is debatable [8]. To solve this problem, various mathematical modeling techniques were proposed to predict the air pollutant concentrations in areas without monitoring stations by extrapolating the monitoring results of the networks. However, as monitoring stations are located at long distances from each other, considerable errors between estimations and actual measurements have been reported [9]. Consequently, a reduction of the distance between stations has been suggested to improve the accuracy of model-based estimations. Unfortunately, since the Korean Peninsula extends southward across different latitudinal climate zones, and 65.2% of the area (South Korea), which corresponds to 64.775 km^2^, is covered by mountainous terrain [10], a large number of air pollution monitoring stations are required to effectively account for these meteorological and topographic characteristics. This negatively affects the economic and practical feasibility. 

The Framework Act on Environmental Policy of South Korea designates sulfur dioxide, carbon monoxide, nitrogen dioxide, fine dust, ozone, lead, and benzene as reference materials of atmospheric pollutants of the environment. Fine dust is classified into PM_10_ and PM_2.5_. Recently, public concern for air pollution caused by fine dust has been increasing and the government is supporting many research projects to develop effective methods of fine dust control. Fine dust is one of the most serious pollutants because it affects air quality, visibility [11], human health [12,13], and global climate change [14]. The effect of fine dust on human health is a global issue and is not limited to South Korea. Many developing countries under industrialization face serious social problems due to fine dust [15,16,17]. Most countries operate air quality monitoring stations to measure fine dust levels in real time. However, PM concentrations measured at a single monitoring point do not accurately represent the fine dust levels to which people around the point are exposed, and it is impossible to analyze the impact of fine dust by using only one measurement device [18]. For this reason, the installation of a high-density network of air quality monitoring stations with high-resolution measurement devices is imperative. However, high-density networks are economically infeasible because they involve large expenses for additional devices. To address this problem, the use of low-cost particle sensors for air quality monitoring and control is attracting increased attention. Considering social and environmental situations in the country as well as abroad, fine dust (PM_10_ and PM_2.5_) has been selected as the target pollutant in this study. 

As a part of the Environmental Policy-Based Public Technology Development Project executed by the Korea Environmental Industry and Technology Institute of the Ministry of Environment, a 32-month research project, which started in November 2016 and will end in June 2019, is underway to set up a smart management support system using sensor technology for pollutant discharge and prevention facilities. For this purpose, this study attempted to solve limitations of the existing method using data from current air pollution monitoring stations for both monitoring air quality and managing discharge and prevention facilities. Moreover, to enhance the reliability of sensor-based measurement devices, this study has derived correction factors by comparing the outputs of domestic sensor-based measurement devices with those of a reference device and succeeded in improving their practicality. The study delivers data that provides a valuable contribution to the abovementioned research project.

## 2. Materials and Methods 

### 2.1. Measurement Devices

Three low-cost, sensor-based measurement devices were tested, which are currently used to measure fine dust levels in South Korea. These devices are manufactured by E3 (Yi Shan A4 sensor, SK techx, Seoul, Korea), MAXFOR Technology (Plantower PMS7003 sensor, SK techx, Seoul, Korea), and SK Techx (Plantower PMS7003 sensor, SK techx, Seoul, Korea). The sensors are referred to as ‘101’, ‘201’, and ‘301’, respectively. Table 1 presents detailed specifications of each device and sensor type. Device 101 is an instrument shelter type with an implemented A4 sensor. Both 201 and 301 use the PMS7003 sensor of Plantower and present the same instrument shelter type as 101. Device 201 is designed to be operated by drawing air in through simple inhalation exposure. 

To compare the trends of output values of the target measurement devices, using cost-effective sensors, regarding concentration interval in real time, a GRIMM (dust spectrometer from Grimm Technologies), based on multi-channel light scattering, was used as the reference device in a real-time test. Before the test, the correction factor (C-factor) of the reference device was calculated using a gravimetric method and by performing an equivalence test. In the gravimetric method, two mini-volume air samplers were used in a chamber of specific dimensions. The C-factor was reflected in the reference device. Particles of Japan Dust Class 10 were used as the test particle for the equivalence test. The details of the equivalence test, including its results, have already been reported by Air Quality Sensor Performance Evaluation Center (AQ-SPEC) [19].

### 2.2. Test Method

The comparison test between the reference device and the target measurement devices using cost-effective particle sensors was performed in the following procedure: The chamber was ventilated using clean air generated by removing particles through a HEPA (high efficiency particulate air) filter (SK techx, Seoul, Korea). After the fine dust level was maintained at or below 2 µg/m^3^ in the chamber, clean air was injected and the ejection was stopped. Once the air flow was at a steady state, both the reference and target devices were operated. The temperature and relative humidity in the chamber were maintained at 20 °C and 40%, respectively. The measurement interval was set to 1 min. The standard powder (Japan Dust) was injected into the chamber and maintained at a certain level. Under this condition, the output values of the reference and target devices were obtained. The test was carried out by classifying the fine dust (PM_2.5_) concentrations into five intervals: 40 µg/m^3^ and below (1st interval), 40–80 µg/m^3^ (2nd interval), 80–120 µg/m^3^ (3rd interval), 120–160 µg/m^3^ (4th interval), and 200–230 µg/m^3^ (5th interval). The measurement was conducted for 150 min for each concentration interval to acquire the values of PM_2.5_ and PM_10_. The obtained output values were utilized for data analysis. 

### 2.3. Evaluation Method

For the output values of each device, descriptive statistics were initially calculated for each concentration interval. To correct the output values of the low-cost, sensor-based measurement devices, a linear regression analysis was conducted by classifying fine dust into PM_2.5_ and PM_10_. In each interval of PM_2.5_ and PM_10_, 75% of the data obtained were randomly extracted, and a linear regression equation was derived using the output values of the GRIMM device as dependent variables and those of the target devices as independent variables. The equation was validated by using the remaining 25% output values, which were not used to derive the equation. 

The precision of a measurement device using a particle sensor should be evaluated based on accuracy and repeatability [20]. Accuracy is the closeness between output values of the target device and measurements of the reference device. Repeatability means dispersion [21]. As there was no correction curve for output values of the target devices, this study utilized linear correlations derived from the linear regression equation to evaluate the accuracy of the devices. In other words, when the output value of a target device was lower or upper than that of the reference device, the accuracy was evaluated to be low. Repeatability was identified based on the variation of the output value of a target device for each particle concentration interval. Finally, the correction factors of each target device, which were calculated using the linear regression equation, were applied to correct the remaining 25% of output values, which had not been used to derive the equation, and the results were compared with the output values of the reference device to evaluate the accuracy of each device under test. In other words, residuals between the output values of the reference device and the corrected values of each sensor-based device were obtained and then divided by the output values of the reference device. The average absolute value thus calculated was used to evaluate the accuracy of each sensor-based device after correction.

## 3. Results and Discussion

### 3.1. PM_2.5_

Table 2 presents descriptive statistics of the output values of the reference device and the sensor-based devices for each PM concentration interval. For every interval, the output values of the reference device were higher than those of the target devices. This indicates that, if the target devices were used without correcting their output values, environmental pollution by PM_2.5_ could be underestimated. Therefore, it was necessary to calculate an appropriate upward correction factor for each device and to evaluate the applicability of the devices after correction. 

Among the target devices, 201 showed the highest values of PM concentrations except for the 5th interval, followed by 101 and 301. In the 5th interval, 101 showed the highest output values, followed by 201 and 301. The output values of 201, which were generally higher than those of the other two devices, showed a similar increasing trend to that of the reference device from the 1st to 4th intervals but decreased in the 5th interval. Like the reference device, 101 showed an increase in output value for all the intervals; 301 also showed a similar increase except for the 4th interval. Accordingly, it appears sufficient to correct the output values of the target devices for each concentration interval instead of the entire concentration range. In addition, as 101 showed a more similarly increasing pattern to the reference device than did 201 and 301, along with the increase of concentration interval, it was evident that 101 maintained a stable sensor sensitivity for each concentration interval whereas the other devices (201 and 301) did not show a constant sensitivity across the intervals. This emphasizes the need to correct the output values of the cost-effective target devices for each concentration interval.

Table 3 presents the results of the linear regression analysis that was conducted by randomly selecting 75% of the data in each concentration interval and using the output values of the reference device as dependent variables and those of 101, 201, and 301 as independent variables. The results show correction factors and correction functions of each device for each interval. Table 4 shows the relative standard deviations which were obtained by calculating residuals between the output values of the reference device and the corrected values of each target device from the remaining 25% of the data, subsequently dividing the residuals by the output values of the reference device. 

For 101, the correction functions of each concentration interval were 4.231x − 1.451 (1st interval), 1.212x + 49.447 (2nd interval), 0.161x + 105.825 (3rd interval), 0.276x + 33.960 (4tht interval), and 1.841x + 115.045 (5th interval) (x = PM_10_ measurement value of each interval). The correction functions thus obtained were used to correct output values for each concentration interval. The relative standard deviations with respect to the output values of the reference device were calculated to be 6.7%, 0.6%, 0.3%, 1.7%, and 1.4% for the respective intervals. All deviations were below 10% and, except for the 1st interval, the deviations in the remaining intervals were even below 5%. This means that when 101 was corrected by the correction functions obtained in this study, its measurements of PM_2.5_ concentration in the real atmosphere featured an error rate of less than 10%. Moreover, for PM_2.5_ concentrations above 40 µg/m^3^, the accuracy of 101 improved with a relative standard deviation of less than 2%. 

For 201, the correction functions for each concentration interval were −0.364x + 41.098 (1st interval), −0.634x + 92.372 (2nd interval), formula 0.246x + 101.604 (3rd interval), 0.202x + 135.458 (4th interval), and 2.465x + 113.471 (5th interval). Based on these correction functions, the relative standard deviations were calculated to be 16.1%, 2.7%, 0.5%, 1.6%, and 0.7% for the respective intervals. Like 101, 201 showed an improvement of accuracy with a relative standard deviation of less than 3% for PM_2.5_ concentrations exceeding 40 µg/m^3^. However, for PM_2.5_ concentrations below 40 µg/m^3^, the accuracy was less reliable. 

For 301, the correction functions for each concentration interval were 6.788x + 11.208 (1st interval), −1.379x + 80.367 (2nd interval), f−0.221x + 111.945 (3rd interval), 0.167x + 141.921 (4th interval), and 1.931x + 178.141 (5th interval). The relative standard deviations were calculated as 10%, 3.3%, 0.5%, 1.8%, and 1.1% for the respective intervals. As for 101 and 201, the PM_2.5_ concentrations measured for 301 in the real atmosphere had an error rate of less than 10%. 301 also displayed an improvement of accuracy with a relative standard deviation of less than 4% for PM_2.5_ concentrations exceeding 40 µg/m^3^. 

All the target measurement devices exhibited relative standard deviations of less than 10% with respect to the measurements of the reference device. When the level exceeded 40 µg/m^3^, their relative standard deviations were less than 4%, indicating an improvement of accuracy. Consequently, the measurement devices using cheap sensors are suitable for monitoring PM_2.5_ levels in the air. In addition, their reliability is enhanced in areas or regions with PM_2.5_ levels exceeding 40 µg/m^3^. Finally, among the target devices, 101 had the highest accuracy.

### 3.2. PM_10_

Table 5 presents descriptive statistics of PM_10_ outputs of the reference device and the target devices for each concentration interval that were set based on PM_2.5_ levels. For every interval, like PM_2.5_, the output values of the reference device were higher than those of the target devices. This shows that, if the sensor-based devices were used without correcting their output values, environmental pollution by PM_10_ could also be underestimated. Accordingly, it was necessary to calculate an appropriate upward correction factor for each device and to evaluate the reliability of the device after correction. 

Among the three sensor-based devices, 201 showed the highest PM concentration values except for the 5th interval, followed by 101 and 301. In the 5th interval, 101 had the highest output values, followed by 201 and 301. The output values of 201, which were higher than those of the other two devices, showed a similarly increasing trend to that of the reference device for the 1st to 4th intervals but decreased in the 5th interval. Like the reference device, 101 showed an increase of output values for all intervals; 301 showed a similar increase except for the 4th interval. The same conclusion as or PM_2.5_ can be indirectly drawn, namely that the output values of the sensor-based devices needed to be corrected for each concentration interval rather than for the whole concentration range. In addition, as 101 showed a more similar pattern to the reference device than did 201 and 301 regarding increasing concentration intervals, it was evident that, unlike the other devices (201 and 301), 101 maintained a stable sensor sensitivity across all concentration intervals. This further emphasizes the need to correct the output values of the target devices for each concentration interval.

Table 6 presents the results of the linear regression analysis using 75% of the PM_10_ data with the output values of the reference and target devices as dependent and independent variables, respectively. Table 7 shows the relative standard deviations, which were obtained by calculating residuals between the output values of the reference device and the corrected values of each sensor-based device from the remaining 25% of the data, subsequently dividing the residuals by the output values of the reference device. 

For 101, the correction functions of each concentration interval were 8.055x − 11.983 (1st interval), 2.453x + 127.747 (2nd interval), 3.296x + 180.915 (3rd interval), 2.368x + 284.705 (4th interval), and 4.248x + 293.755 (5th interval) (x = PM_2.5_ measurement value of each section). The correction functions thus obtained were used to correct output values for each concentration interval. The relative standard deviations with respect to the output values of the reference device were calculated to be 9.8%, 3.2%, 0.9%, 1.7%, and 1.2% in the respective intervals. All the deviations were below 10% and, except for the 1st interval, the deviations were even below 4%. This shows that when 101 is corrected by the correction functions obtained in this study, measurements of PM_10_ concentration in the real atmosphere feature an error rate of less than 10%. Moreover, for PM_10_ concentrations above 40 µg/m^3^, the accuracy of 101 improved with a relative standard deviation of less than 4%. 

For 201, the correction functions of each concentration interval were 1.583x + 14.320 (1st interval), −1.368x + 243.022 (2nd interval), 1.139x + 253.129 (3rd interval), 1.172x + 337.257 (4th interval), and 3.920x + 385.647 (5th interval). Based on these correction functions, the relative standard deviations were calculated to be 22.7%, 3.0%, 1.6%, 1.6%, and 0.8% in the respective intervals. Like 101, 201 showed an improvement of accuracy with a relative standard deviation of less than 3% for PM_10_ concentrations exceeding 40 µg/m^3^. On the other hand, for PM_10_ concentrations below 40 µg/m^3^, the accuracy was diminished. 

For 301, the correction functions for each concentration interval were 13.713x + 1.475 (1st interval), −2.586x + 213.285 (2nd interval), 2.941x + 269.541 (3rd interval), −0.892x + 405.137 (4th interval), and 2.147x + 523.669 (5th interval). The relative standard deviations were calculated to be 21.2%, 4.1%, 1.3%, 1.8%, and 0.9% for the respective intervals. Like 101 and 201, the measurements of 301 featured a low accuracy for PM_10_ concentrations below 40 µg/m^3^ in the real atmosphere. However, 301 also displayed an improvement of accuracy with a relative standard deviation of less than 4.5% for PM_2.5_ concentrations above 40 µg/m^3^. 

As for PM_2.5_, all target devices showed low accuracy with relative standard deviations exceeding 10% for PM_10_ levels below 40 µg/m^3^. However, when the level exceeded 40 µg/m^3^, the relative standard deviations decreased to less than 5%, indicating an improvement of accuracy. Consequently, the target measurement devices are suitable for monitoring PM_10_ levels in areas or regions with PM_10_ levels exceeding 40 µg/m^3^. Among all target devices, 101 had the highest accuracy for PM_10_ measurements.

## 4. Conclusions

As part of a government-sponsored research and development project to establish a smart management support system for pollutant discharge and prevention facilities, this study evaluated the reliability of sensor-based measurement devices to overcome limitations of existing methods. To this end, data from air pollution stations for monitoring air quality and managing discharge and prevention facilities were analyzed. Also, correction functions were derived by comparing cost-effective sensor-based measurement devices with a reference device, and the accuracy of corrected values, which were obtained by applying the functions, was examined.

The low-cost sensor uses the light scattering method for measurement and thus is lower in cost than the gravimetric of beta ray absorption devices. Moreover, as the low-cost sensor not only can measure fine dust levels on a minute time basis but is also small and light, it can be made in both a fixed type and portable type. However, this sensor tends to produce over- or under-measurements compared to other methods and the densities of fine dust emissions from various sources are not the same. For this reason, an error often occurs while the amount of scattering light is converted to a mass concentration. A correction formula has been developed to reduce such errors. If the correction formula is applied, as the proposed method is less expensive than other methods, the distances between each station may be reduced at a lower cost.

The evaluation showed that, under the condition of PM_2.5_ concentrations exceeding 40 µg/m^3^, all the target devices (101, 201, and 301) had a relative standard deviation of less than 5% for both corrected PM_2.5_ and PM_10_ levels using the correction functions. It is postulated that most facilities involve PM_2.5_ concentrations exceeding 40 µg/m^3^. In this regard, the above cost-effective sensor-based measurement devices are more reliable than the numerical analysis-based monitoring method. The conventional monitoring method uses data from national air pollution monitoring stations from discharge and prevention facilities at a long distance from each other. Therefore, data from such stations may not be very trustworthy for monitoring and controlling fine dust levels. Instead, sensor-based measurement devices, which are cost effective and easy to implement, may improve the development of a smart management support system for pollutant discharge and prevention facilities.

## Figures and Tables

**Table 1:** Specifications of measuring devices.

Model	GRIMM	E3	MAXFOR	SK TechX
Abbreviation used in this work	GRIMM	101	201	301
Appearance	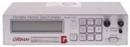	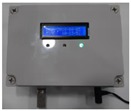	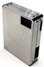	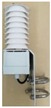
Measurement Range	0.25–32 µm in 31 size channels	0–6000 µg/m^3^	0–500 µg/m^3^	0–500 µg/m^3^
Temperature range	0–40 °C	−20–70 °C	−40–125 °C	−10–60 °C
Humidity range	0–95% RH (non-condensing)	0–100% RH	0–100% RH	0–99% RH
Wind direction	-	0–360°	0–360°	-
Wind speed	-	0.5–89 m/s	0.5–89 m/s	-
Sensor manufacturer	GRIMM	Yi Shan	Plantower	Plantower

**Table 2:** Comparison of descriptive statistics of PM_2.5_ output according to the concentration ranges of the measuring devices.

Devices Section		GRIMM	101	201	301
Mean (µg/m³)	1st Interval	30.30	7.58	29.26	2.80
	2nd Interval	71.38	18.05	32.36	6.09
	3rd Interval	110.16	26.88	34.92	7.84
	4th Interval	143.94	33.56	38.62	7.55
	5th Interval	197.43	45.11	34.18	10.24
SD	1st Interval	11.28	9.84	1.59	8.13
	2nd Interval	4.44	4.24	3.60	9.74
	3rd Interval	4.74	6.46	7.11	10.74
	4th Interval	3.83	5.87	9.18	19.10
	5th Interval	7.87	9.82	11.34	25.74
Medium (µg/m³)	1st Interval	27.4	7	29	2.7
	2nd Interval	71.0	18	32	6.0
	3rd Interval	110.3	27	35	7.8
	4th Interval	144.2	33	39	7.5
	5th Interval	197.7	45	34	10.1

**Table 3:** Result of regression analysis of 75% of PM_2.5_ output for each sensor.

Model		B	Standard Error	95% Confidence Interval		*p*-Value
				Lower Bounding	Upper Bounding	
1st Interval	(Constant)	−1.451	1.853	−4.411	3.181	0.437
	101	4.231	0.233	3.609	4.535	0.000
	(Constant)	41.098	27.360	−13.630	95.826	0.138
	201	−0.364	0.936	−2.237	1.509	0.699
	(Constant)	11.208	4.717	1.773	20.644	0.021
	301	6.788	1.597	3.592	9.983	0.000
2nd Interval	(Constant)	49.447	10.326	28.624	70.271	0.000
	101	1.212	0.573	0.056	2.368	0.040
	(Constant)	92.372	13.315	65.501	119.243	0.000
	201	−0.634	0.411	−1.464	0.197	0.131
	(Constant)	80.367	4.686	70.910	89.824	0.000
	301	−1.379	0.755	−2.902	0.143	0.075
3rd Interval	(Constant)	105.825	14.804	75.927	135.723	0.000
	101	0.161	0.546	−0.941	1.264	0.769
	(Constant)	101.604	9.603	82.209	120.998	0.000
	201	0.246	0.275	−0.309	0.802	0.375
	(Constant)	111.945	6.517	98.785	125.106	0.000
	301	−0.221	0.820	−1.878	1.435	0.789
4th Interval	(Constant)	133.960	13.278	107.164	160.755	0.000
	101	0.276	0.396	−0.523	1.075	0.490
	(Constant)	135.458	8.186	118.938	151.977	0.000
	201	0.202	0.213	−0.228	0.632	0.348
	(Constant)	141.921	3.925	134.000	149.842	0.000
	301	0.167	0.504	−0.850	1.184	0.742
5th Interval	(Constant)	115.045	17.295	80.141	149.948	0.000
	101	1.841	0.383	1.068	2.614	0.000
	(Constant)	113.471	12.514	88.217	138.725	0.000
	201	2.465	0.364	1.730	3.199	0.000
	(Constant)	178.141	7.346	163.316	192.966	0.000
	301	1.931	0.706	0.506	3.355	0.009

**Table 4:** Results of the relative standard deviation of PM_2.5_ output for each sensor after 25% calibration.

Model		Minimum Value	Maximum Value	Mean	Absolute Value of Mean
1st Interval	101	−0.47	0.17	−0.0669	0.0669
	201	−1.31	0.45	−0.1609	0.1609
	301	−1.10	0.37	−0.0975	0.0975
2nd Interval	101	−0.11	0.14	−0.0006	0.0006
	201	−0.13	0.07	−0.0274	0.0274
	301	−0.14	0.08	−0.0328	0.0328
3rd Interval	101	−0.13	0.09	−0.0029	0.0029
	201	−0.14	0.09	−0.0045	0.0045
	301	−0.14	0.10	−0.0047	0.0047
4th Interval	101	−0.03	0.08	0.0169	0.0169
	201	−0.03	0.08	0.0158	0.0158
	301	−0.03	0.08	0.0178	0.0178
5th Interval	101	−0.06	0.06	−0.0136	0.0136
	201	−0.07	0.05	−0.0065	0.0065
	301	−0.07	0.06	−0.0105	0.0105

**Table 5:** Comparison of descriptive statistics of PM_10_ output according to the concentration ranges of measuring devices.

Devices Section		GRIMM	101	201	301
Mean (µg/m³)	1st Interval	68.84	10.19	35.07	5.03
	2nd Interval	187.38	24.87	39.74	9.31
	3rd Interval	303.12	37.24	44.78	11.68
	4th Interval	396.97	46.57	49.34	11.47
	5th Interval	556.26	62.16	43.79	15.69
SD	1st Interval	29.68	3.36	2.12	1.07
	2nd Interval	14.61	1.51	3.15	1.22
	3rd Interval	14.53	2.18	2.91	1.40
	4th Interval	13.07	1.83	3.64	1.61
	5th Interval	22.10	2.62	3.23	2.23
Medium	1st Interval	61	9	35	5
	2nd Interval	189	25	40	9
	3rd Interval	302	38	45	12
	4th Interval	399	46	50	12
	5th Interval	559	63	44	16

**Table 6:** Results of regression analysis of 75% of PM_10_ output for each sensor.

Model		B	Standard Error	95% Confidence Interval		*p*-Value
				Lower Bounding	Upper Bounding	
1st Interval	(Constant)	−11.983	5.229	−22.443	−1.523	0.025
	101	8.055	0.488	7.078	9.031	0.000
	(Constant)	14.320	62.591	−110.879	139.520	0.820
	201	1.583	1.781	−1.979	5.145	0.377
	(Constant)	1.475	15.922	−30.374	33.323	0.927
	301	13.713	3.120	7.473	19.953	0.000
2nd Interval	(Constant)	127.747	38.773	49.499	205.995	0.002
	101	2.453	1.553	−0.680	5.586	0.122
	(Constant)	243.022	29.453	183.584	302.460	0.000
	201	−1.368	0.742	−2.866	0.130	0.072
	(Constant)	213.285	17.403	178.165	248.405	0.000
	301	−2.586	1.831	−6.280	1.109	0.165
3rd Interval	(Constant)	180.915	31.141	118.024	243.806	0.000
	101	3.296	0.838	1.604	4.987	0.000
	(Constant)	253.129	31.660	189.190	317.068	0.000
	201	1.139	0.704	−0.283	2.561	0.113
	(Constant)	269.541	18.087	233.013	306.069	0.000
	301	2.941	1.524	−0.137	6.018	0.061
4th Interval	(Constant)	284.705	47.801	188.238	381.172	0.000
	101	2.368	1.027	0.295	4.441	0.026
	(Constant)	337.257	25.764	285.264	389.250	0.000
	201	1.172	0.523	0.116	2.227	0.030
	(Constant)	405.137	13.296	378.306	431.969	0.000
	301	−0.892	1.138	−3.188	1.404	0.437
5th Interval	(Constant)	293.755	63.627	165.350	422.160	0.000
	101	4.248	1.023	2.182	6.313	0.000
	(Constant)	385.647	33.516	318.009	453.285	0.000
	201	3.920	0.762	2.383	5.458	0.000
	(Constant)	523.669	20.202	482.901	564.438	0.000
	301	2.147	1.263	−0.403	4.697	0.097

**Table 7:** Results of the relative standard deviation of PM_10_ output for each sensor after 25% calibration.

Model		Minimum Value	Maximum Value	Mean	Absolute Value of Mean
1st Interval	101	−0.36	0.18	−0.0977	0.0977
	201	−1.36	0.52	−0.2271	0.2271
	301	−1.08	0.30	−0.2119	0.2119
2nd Interval	101	−0.13	0.14	−0.0317	0.0317
	201	−0.16	0.11	−0.0301	0.0301
	301	−0.15	0.12	−0.0405	0.0405
3rd Interval	101	−0.15	0.07	−0.0091	0.0091
	201	−0.13	0.07	−0.0158	0.0158
	301	−0.11	0.08	−0.0130	0.0130
4th Interval	101	−0.02	0.06	0.0168	0.0168
	201	−0.03	0.08	0.0161	0.0161
	301	−0.04	0.08	0.0175	0.0175
5th Interval	101	−0.06	0.06	−0.0118	0.0118
	201	−0.07	0.06	−0.0084	0.0084
	301	−0.07	0.07	−0.0091	0.0091
